# Extracellular Vesicle Phenotypic Trajectories During Alcohol Withdrawal Treatment

**DOI:** 10.3390/ijms27156593

**Published:** 2026-07-24

**Authors:** Vsevolod V. Severtsev, Alexander A. Yakovlev, Alexey S. Golovkin, Arthur D. Aquino, Adrian A. Zavodov, Vasiliy S. Chernyshev, Maria A. Vinnikova, Yuliya G. Tikhonova, Milausha Sh. Galeeva, Marina A. Kinkulkina, Nikolay N. Ivanets

**Affiliations:** 1 Department of Psychiatry and Narcology, N.V. Sklifosovsky Institute of Clinical Medicine, Sechenov University, 119048 Moscow, Russia; vinnikova_m_a@staff.sechenov.ru (M.A.V.); tikhonova_yu_g@staff.sechenov.ru (Y.G.T.); mil5119@yandex.ru (M.S.G.); kinkulkina_m_a@staff.sechenov.ru (M.A.K.); ivanets_n_n@staff.sechenov.ru (N.N.I.); 2 Laboratory of Cellular Neurobiology of Learning, Institute of Higher Nervous Activity and Neurophysiology, Russian Academy of Sciences, 117485 Moscow, Russia; al_yakovlev@ihna.ru; 3 Laboratory of Microvesicular Signaling, Institute of Molecular Biology and Genetics, Almazov National Medical Research Center, 197341 St. Petersburg, Russia; golovkin_a@mail.ru (A.S.G.); akino97@bk.ru (A.D.A.); 4 Skolkovo Innovation Center, Skolkovo Institute of Science and Technology, 121205 Moscow, Russia; a.zavodov@skoltech.ru (A.A.Z.); v.chernyshev@skoltech.ru (V.S.C.); 5 National Medical Research Center for Obstetrics, Gynecology and Perinatology Named After Academician V.I. Kulakov, Ministry of Healthcare of the Russian Federation, 117997 Moscow, Russia; 6 Moscow Research and Practical Center for Narcology of the Moscow Department of Health, 109390 Moscow, Russia

**Keywords:** alcohol withdrawal syndrome, extracellular vesicles, temporal dynamics, vesicle morphology, vesicle phenotypes

## Abstract

Alcohol dependence or alcohol use disorder is a chronic mental disorder that imposes a heavy global burden. Small extracellular vesicles (EVs) are emerging as a promising source of real-time information about brain status, yet existing studies are predominantly cross-sectional. We aimed to characterize longitudinal changes in EV properties and immunophenotype during alcohol withdrawal treatment. In our study plasma-derived EVs were analyzed and immunophenotyping via flow cytometry was performed to measure the concentrations of vesicle fractions carrying erythrocyte-, platelet-, endothelial-, and neuronal-associated markers in patients undergoing alcohol withdrawal and in healthy volunteers. Patient-derived EVs exhibited greater size heterogeneity, and microscopy images revealed vesicles with altered morphology. Concentrations of EVs bearing NLGN3 were lower in patients. CD62P+ and CD146+ EVs were also less represented in patients. Notably, CD31+ EVs separated into two distinct clusters; their concentrations were higher in patients with a binge drinking pattern but markedly lower in those with a steady pattern. The findings reveal dynamic phenotypic shifts in circulating EVs during alcohol withdrawal. The divergent patterns according to alcohol use history highlight the potential of EV profiling for personalized monitoring and underscore the necessity of longitudinal designs in vesicle-based biomarker research.

## 1. Introduction

Alcohol dependence or alcohol use disorder is a chronic, progressive mental disorder. As of 2019, an estimated 209 million individuals over the age of 15 were living with alcohol dependence globally [1]. Unlike other mental disorders, alcohol dependence results from an interplay of chronic intoxication, personal physiological makeup, and advancing somatic dysfunction.

Small extracellular vesicles (sEVs) are membrane-bound vesicles up to 200 nm in size. They represent one mode of intercellular communication, including between the brain and organs located beyond the blood–brain barrier [2]. Cells package various signaling molecules into sEVs, such as proteins, cytokines, and nucleic acids, and also leave membrane proteins on their surface that serve as markers of cellular origin [2]. In the context of addictive disorders, sEVs are of particular interest because, on the one hand, they allow minimally invasive, “real-time” study of processes occurring in the central nervous system; on the other hand, they enable detection of signals that organs send to each other, including to the brain [3].

However, the field is increasingly challenged by the substantial and often underestimated heterogeneity of these nanoparticles, which encompasses a spectrum of subtypes, from exosomes and microvesicles to apoptotic bodies, each defined by distinct biogenetic pathways—including endosomal sorting complexes required for transport (ESCRT)-dependent and -independent mechanisms—and variable biophysical properties [2,4]. This inherent complexity is further compounded by methodological challenges in the field, as current isolation techniques, such as ultracentrifugation and size-exclusion chromatography, and detection methods, including nanoparticle tracking analysis and flow cytometry, exhibit significant trade-offs in purity, yield, and scalability, complicating data interpretation and cross-study comparisons [2,5]. Crucially, the heterogeneity of EV populations is not merely a technical nuisance but a biologically significant phenomenon, arising from differences in cellular origin, donor age, sex, disease status, and treatment conditions [6,7]. This variability has profound implications, as it can profoundly influence the diagnostic accuracy of EV-based biomarkers and dictate the therapeutic efficacy and safety of EV-based interventions, underscoring the urgent need for standardized characterization frameworks and a deeper mechanistic understanding of how these variables shape EV identity and function across different biological contexts [8].

In recent years, interest in sEVs in the context of addictive disorders has been steadily growing, although this field is relatively young [9,10]. To date, it has been shown that during acute and chronic alcohol intoxication, the expression of various microRNAs and proteins in sEVs changes [11,12,13,14]; the lipid composition of sEVs in both blood and urine shows sex-dependent alterations [15,16,17]; and the distribution of cytokines packaged in sEVs versus those freely circulating in plasma also changes [18]. Nevertheless, the studies described are cross-sectional, which does not allow definitive conclusions regarding the stability of the observed changes over time or the potential use of such findings as biomarkers or targets for pathogenetic treatment. Moreover, the existing studies provide limited clinical data on the participating patients, which significantly narrows the interpretability of the results.

In the present study, we analyzed the representation of different EVs isolates over time in patients during acute alcohol withdrawal syndrome, using flow cytometry based on protein markers.

## 2. Results

### 2.1. Clinical Characteristics

The study involved 28 participants, with 14 individuals in each group. The groups were comparable in terms of sex, age, and body mass index (BMI). The clinical characteristics of the patients are presented in Table 1.

All patients underwent routine laboratory evaluation, including a complete blood count and a biochemical blood panel (Table 2). The measured parameters for the majority of patients fell within the respective reference ranges.

All patients received pharmacotherapy in accordance with current clinical guidelines [19]: benzodiazepines (bromdihydrochlorphenylbenzodiazepine). Thiamine was received by 86% (*n* = 12) of patients, antipsychotics by 86% (*n* = 12), antidepressants by 86% (*n* = 12), and anticonvulsants by 64% (*n* = 9). Treatment resulted in successful relief of withdrawal syndrome in all patients, with no complicated forms observed; all patients were discharged with a motivation for sobriety. Three participants (20%) from the control group were taking oral contraceptives. 

### 2.2. Characterization of Isolated Vesicles

**Figure 1:**
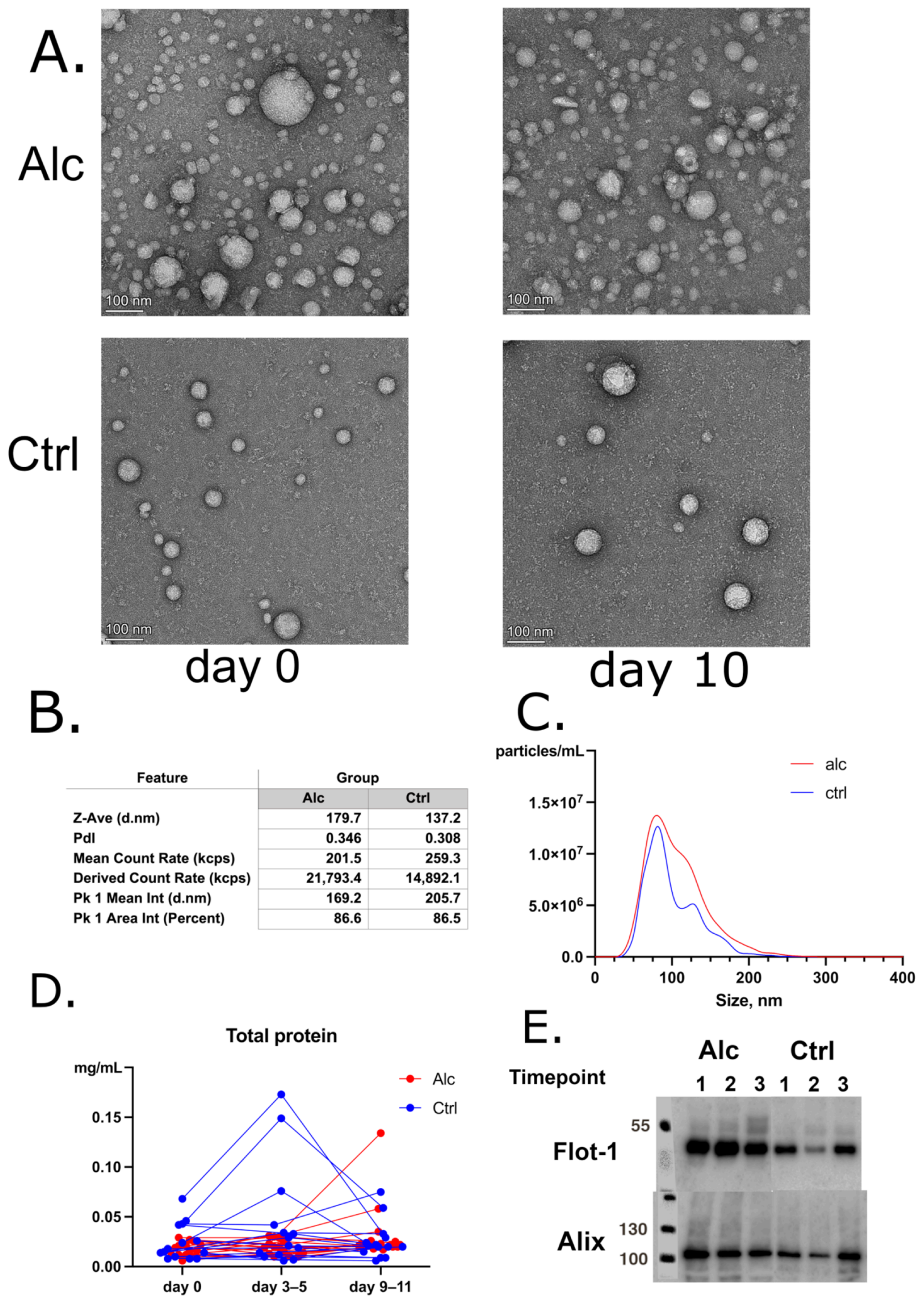
Characteristics of the isolated fraction of extracellular vesicle. Panel (**A**) shows representative microscopy images. Panel (**B**) presents the DLS results, and Panel (**C**) the NTA results. Individual total protein concentrations for each sample are shown in Panel (**D**) with red points for Alcohol group and blue points for control group. Panel (**E**) displays representative immunoblotting results. Z-Ave: Z-average (intensity-weighted mean hydrodynamic diameter); PdI: polydispersity index; Mean Count Rate: mean count rate (kcps); Derived Count Rate: derived count rate (kcps); Pk 1 Mean Int: peak 1 mean intensity (%); Flot-1: flotillin-1; Alix: ALG-2-interacting protein X (PDCD6IP).

**Figure A1:**
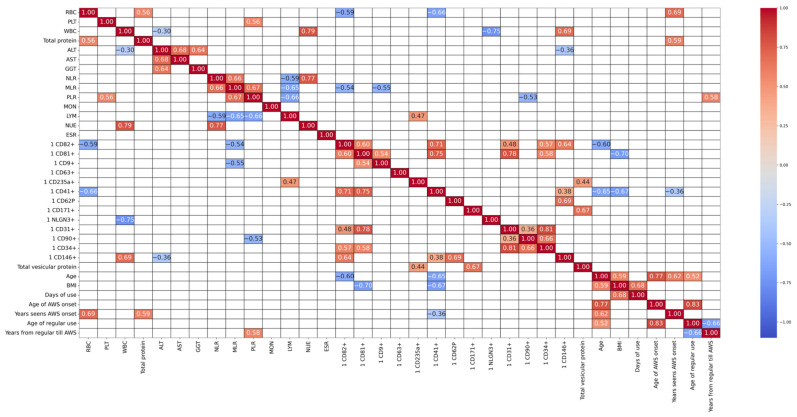
Correlation matrix of the clinical data and the concentrations of vesicle fractions at admission.

According to high-resolution transmission electron microscopy image analysis, the most abundant fraction in all samples consisted of EVs sized 30–40 nm. In the Alcohol group samples, EVs of about 20 nm and 100 nm were also observed, whereas in the control group samples the size remained in the range of 30–90 nm (Figure 1A). Greater morphological heterogeneity and the presence of aggregates were also noticeable in the patient samples. According to dynamic light scattering (DLS) results (Figure 1B), the average vesicle diameter in patients was greater than in the control group, and the polydispersity index was higher in the Alcohol group, which also confirms slightly greater size diversity of vesicles in that group. Nanoparticle tracking analysis (NTA) results (Figure 1C) showed: mean size 106.9 ± 4.8 nm in the Alcohol group with a mean concentration of 7.6 ± 4.5 × 10^10^ particles/mL, and mean size 103.7 ± 11.8 nm in the control group with a mean concentration of 6.3 ± 5.5 × 10^10^ particles/mL, although a “tail” of larger vesicles can be seen in the Alcohol group. No significant differences in total protein concentration were found either between groups or across different time points (Figure 1D); however, protein concentration was characterized by considerable heterogeneity, with a minimum value of 0.006 mg/mL and a maximum of 0.173 mg/mL. Total vesicular protein concentration did not depend on sex, age, or the presence of toxic hepatitis or hepatic steatosis and did not correlate with other clinical characteristics (Appendix A Figure A1). Total vesicular protein concentration on day 0 did not correlate with total blood protein concentration: r_xy_ (95% CI) = 0.384 (−0.184–0.760) (*p* = 0.175). Immunoblotting results (Figure 1E) demonstrated the presence of EV protein markers—Alix and Flotillin-1.

**Figure 2:**
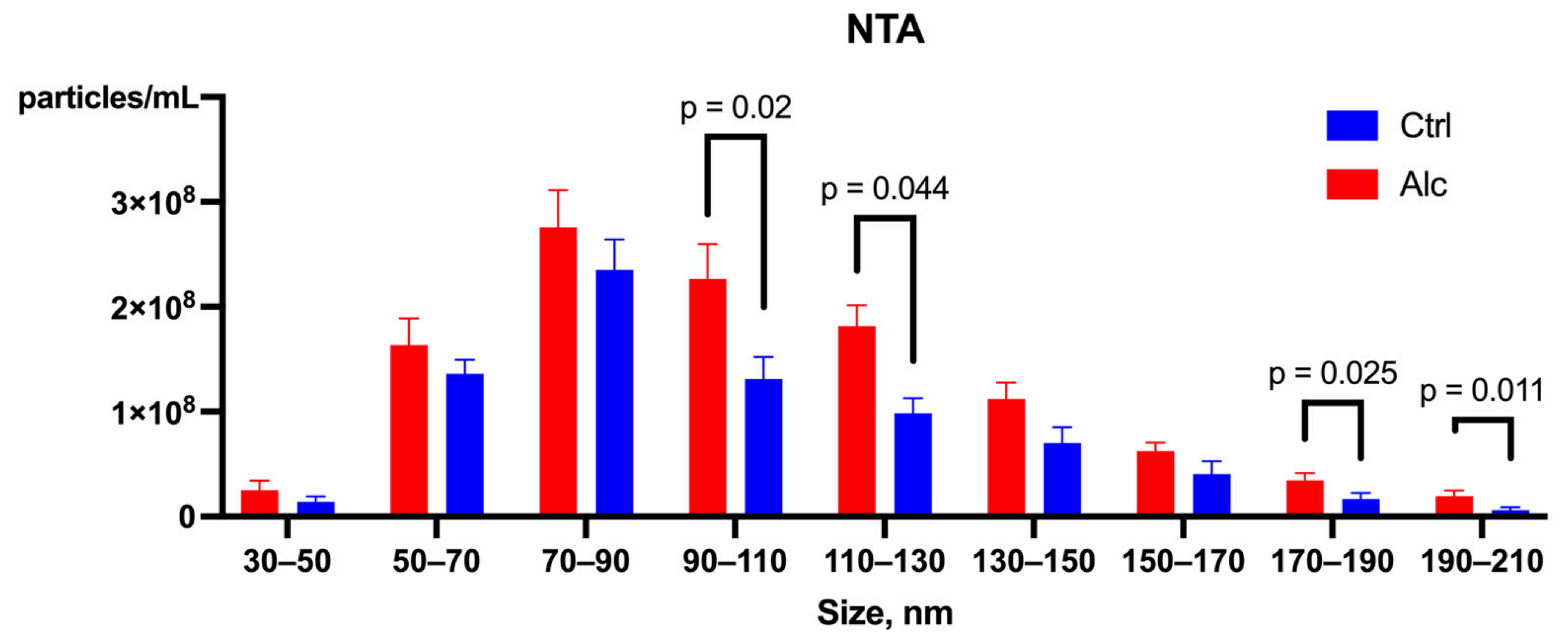
Results of NTA. Red columns—mean concentration with SEM of the Alcohol group; Blue columns—mean concentration with SEM of the control group.

As bulk concentration can mask size-selective changes, we partitioned the particle diameter distribution into 20 nm bins. While the Alcohol group showed a broad trend toward higher counts, the bin-by-bin comparison selectively uncovered statistically significant increases in the 90–130 nm and 170–210 nm size ranges (Figure 2).

### 2.3. Immunophenotyping of EVs

**Figure 3:**
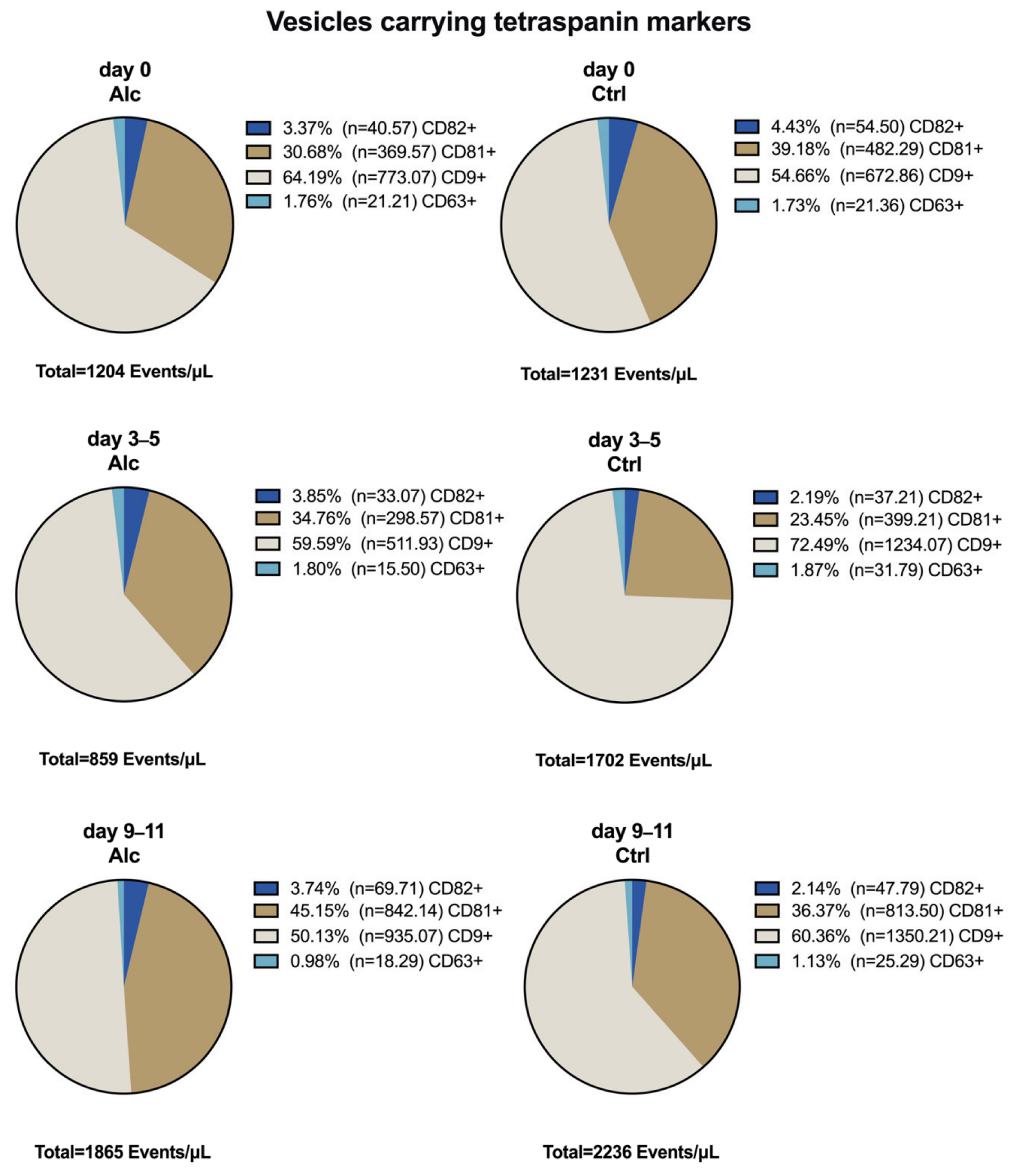
Mean CD9+, CD63+, CD81+, CD82+ vesicles concentrations detected by flow cytometry.

Vesicles carrying the EVs markers CD9, CD81, CD82, and CD63 were detected in all samples. Their concentrations did not differ significantly between the groups at any time point, nor across time points (Figure 3). Vesicles positive for CD9 and CD81 were the most abundant, accounting for approximately 90% of all detected marker-positive vesicles. Of note, the dynamic pattern in the Alcohol group differed from that in healthy volunteers: the proportion of CD9+ vesicles decreased and became comparable to that of CD81+ vesicles, whereas in the control group the CD9+ fraction consistently predominated, reaching 73% at the second time point.

**Figure 4:**
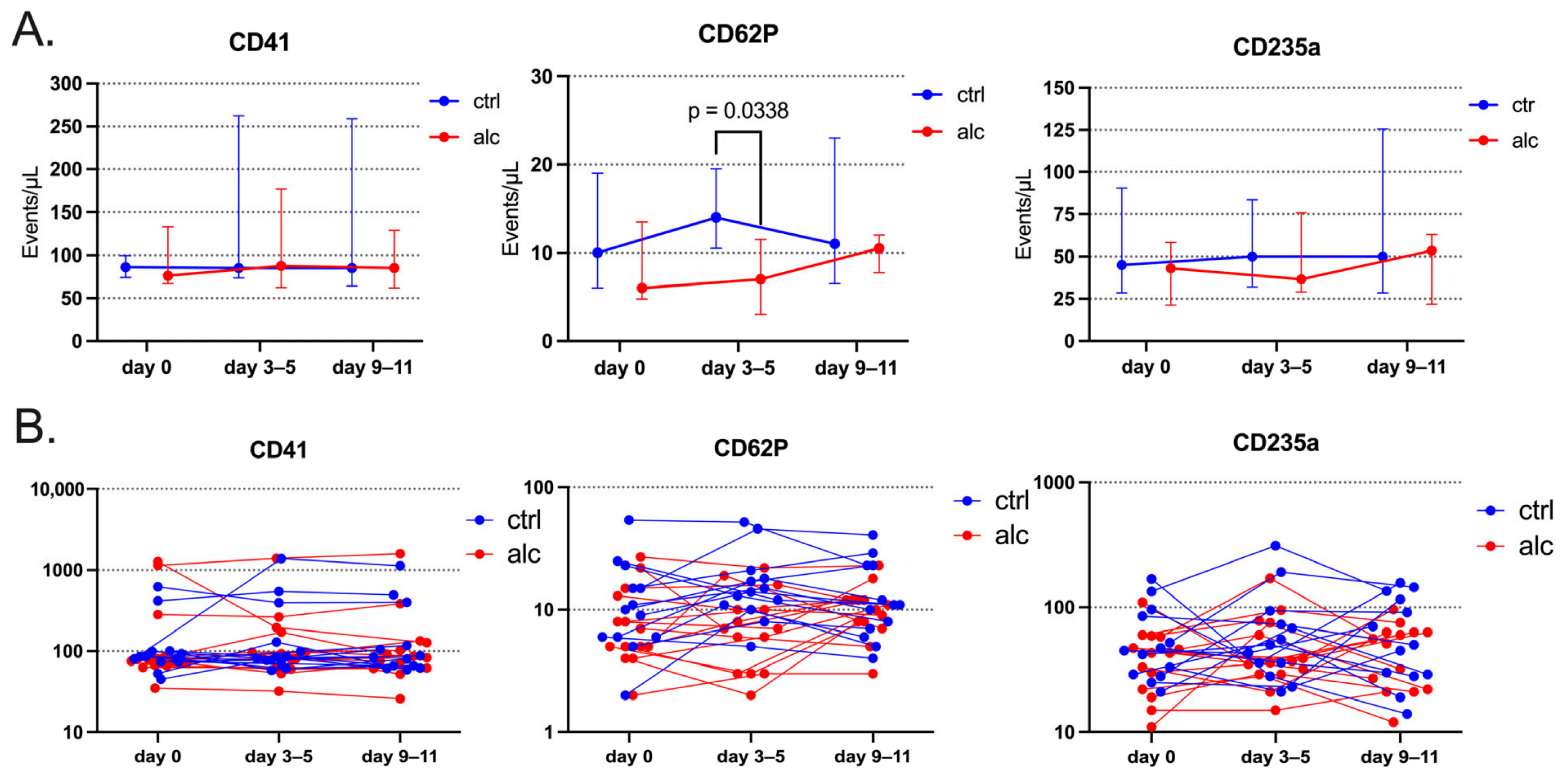
Concentrations of vesicles carrying platelet- and erythrocyte-associated markers. Blue points: median concentration in the control group; red points: median concentration in the Alcohol group. Vertical blue lines: interquartile ranges (IQR) for the control group; vertical red lines: IQR for the Alcohol group. Panel (**A**) displays median and IQR values, while Panel (**B**) shows individual data points.

Differences in EV concentration were observed exclusively at the second time point and solely for the subpopulation positive for CD62P (Figure 4). For vesicles positive for the platelet-associated marker CD41 and the erythrocyte-associated marker CD235a, no significant changes in concentration were detected at any of the time points examined.

The individual-value plots reveal that concentrations of CD62P+ and CD235a+ vesicles in the Alcohol group tended to be lower; however, high variability and significant changes in concentration across time points were evident in both groups. In contrast, CD41+ vesicle concentrations remained stable in the majority of participants.

**Figure 5:**
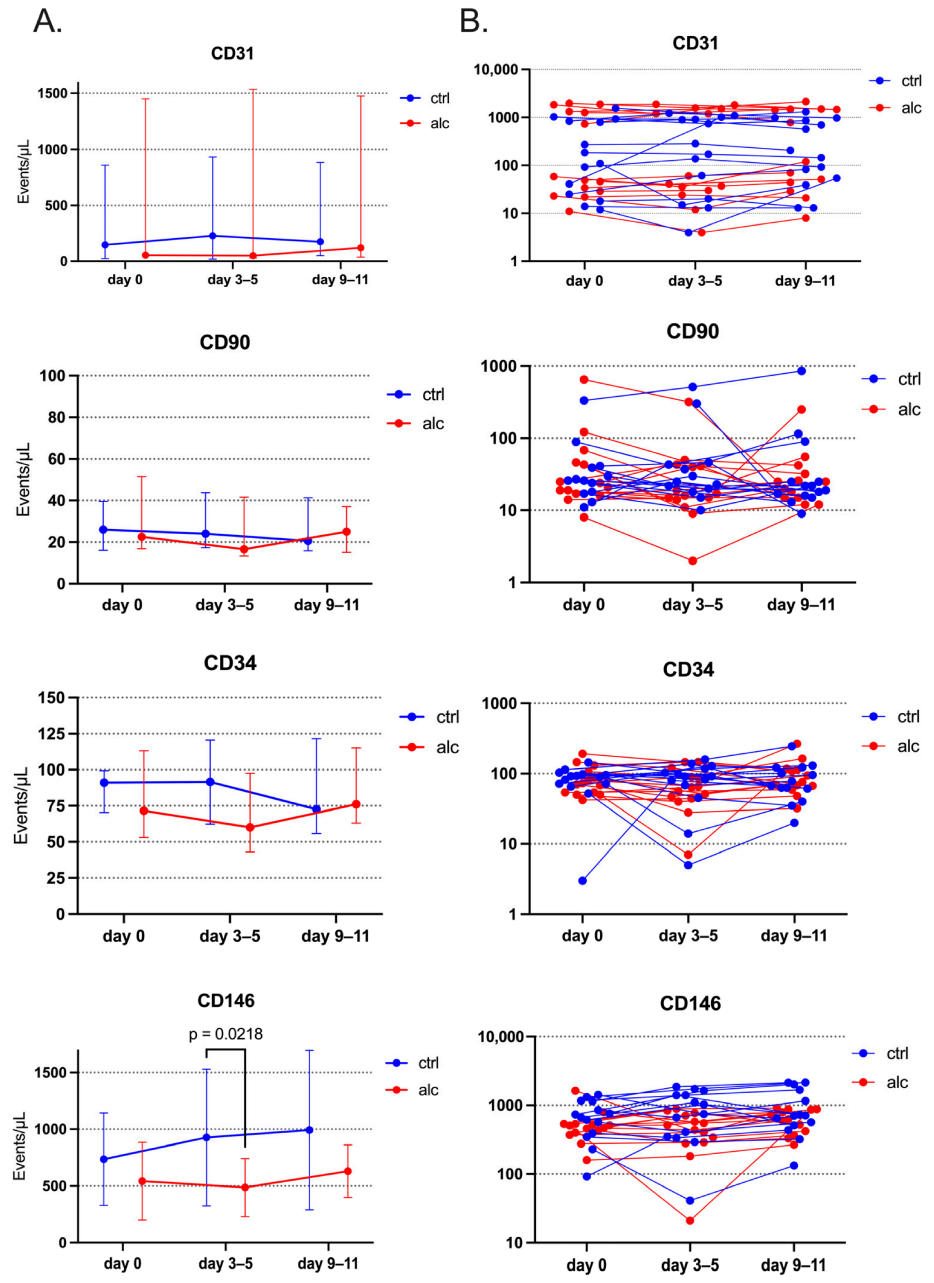
Concentrations of vesicles carrying endothelial cell-associated markers. Blue points: median concentration in the control group; red points: median concentration in the Alcohol group; vertical blue lines: IQR in the control group; vertical red lines: IQR in the Alcohol group. Panel (**A**) (**left**) displays median and IQR values, while Panel (**B**) (**right**) shows individual data points.

As shown in Figure 5, significant changes were recorded exclusively for the concentration of CD146+ Evs. The concentrations of CD31+, CD90+, and CD34+ vesicles were also quantitatively assessed; however, no significant differences were detected for any of the corresponding vesicle subpopulations at any of the time points examined. CD34+ and CD146+ vesicle concentrations tended to be lower in the Alcohol group; notably, CD146+ vesicles showed a statistically significant difference at the 3–5 day time point (Figure 5). Despite this, overall concentrations remained relatively stable across time points in both groups.

**Figure 6:**
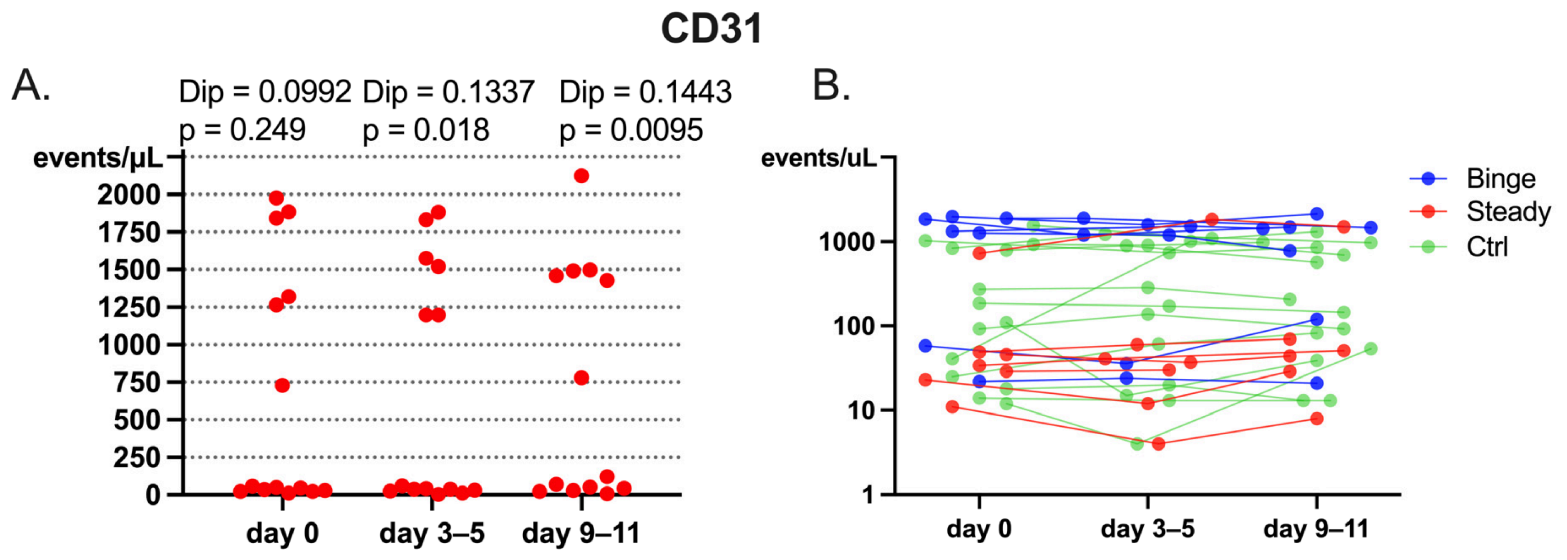
Concentrations of vesicles carrying CD31. Panel (**A**) shows individual CD31+ vesicle concentrations in the Alcohol group. For each time point, the results of Hartigan’s Dip Test are presented to reject the assumption of unimodality, thereby confirming the presence of two clusters. In Panel (**B**): blue points represent individual values for patients with a binge drinking pattern; red points represent individual values for patients with a steady drinking pattern; green points represent individual values for the control group.

Notably, the CD31+ vesicle concentrations in the Alcohol group displayed a markedly wide interquartile range (Figure 5), prompting further exploratory analysis. Stratifying the data by CD31+ vesicle abundance revealed two distinct subgroups—one characterized by high concentrations and the other by low concentrations (Figure 6A). The trough between the two subgroups fell within approximately 100–700 events/µL; therefore, a threshold of 700 events/µL was empirically determined for subsequent exploratory analysis. Integration with clinical phenotypes showed that low CD31+ vesicle levels were associated with a steady drinking pattern, while high levels characterized patients with a binge drinking pattern (Figure 6B).

Although this association did not reach statistical significance (Table 3), it may have remained undetected owing to the limited sample size: 75% of the low CD31+ vesicle concentration subgroup were steady drinkers, whereas 83% of the high concentration subgroup were binge drinkers (*p* = 0.103).

Further analysis revealed that the subgroup with a CD31+ vesicle concentration below 700 events/µL at day 0 exhibited a “low-vesicle” phenotype, with significantly lower concentrations of CD82+, CD81+, and CD41+ throughout the entire observation period, of CD34+ during the first five days, of CD9+ at day 0, and of NLGN3+ at day 10 (Table 3). However, total vesicular protein concentration was comparable between the subgroups. Clinical characteristics did not differ significantly, but a trend towards a later onset of AWS was observed in this subgroup. Moreover, no significant differences were detected in blood test results between the subgroups (Table 3).

**Figure 7:**
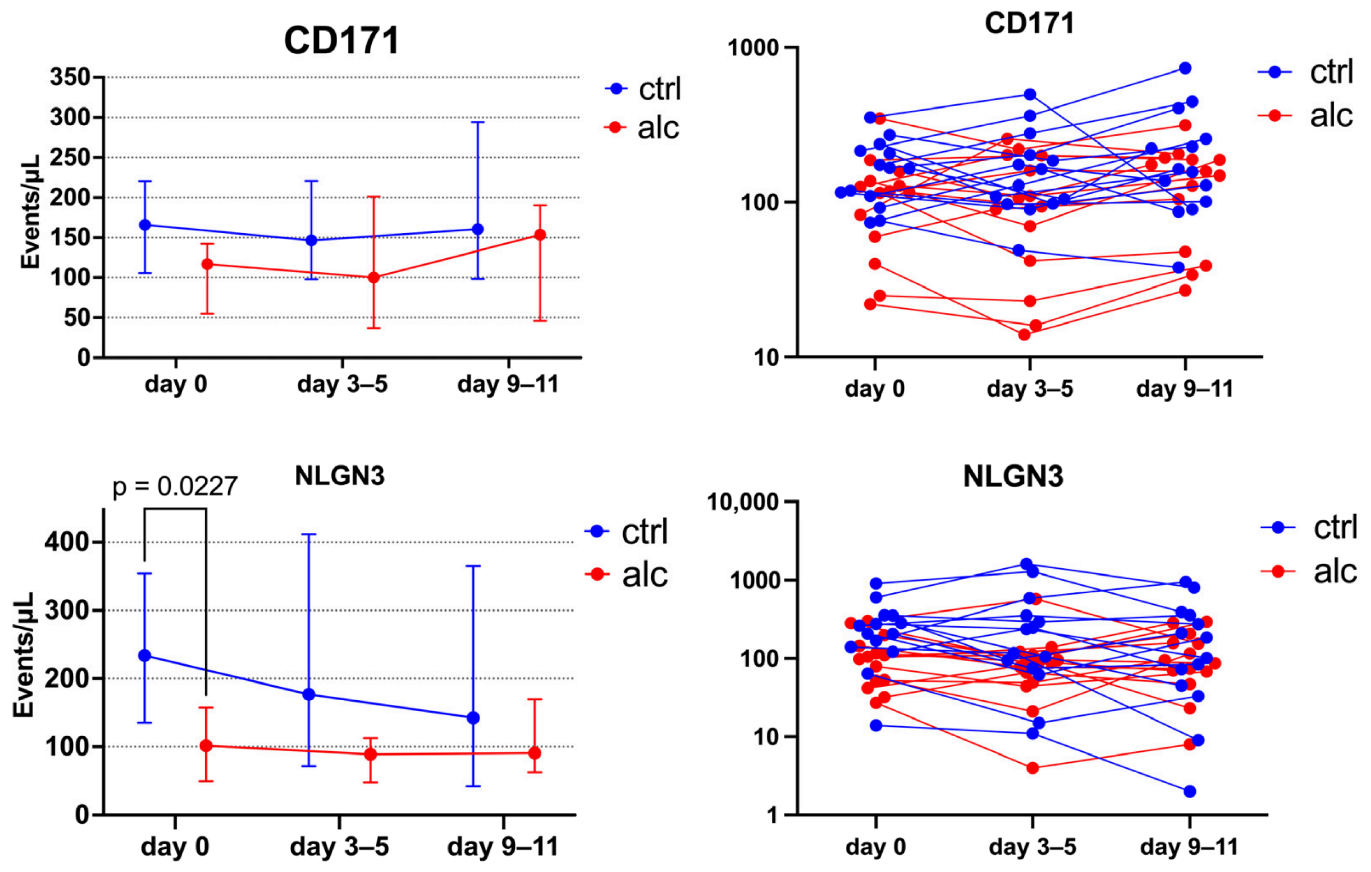
Concentrations of vesicles expressing neuron-associated markers. Blue points: median concentration in the control group; red points: median concentration in the Alcohol group; vertical blue lines: IQR in the control group; vertical red lines: IQR in the Alcohol group. Panel (**left**) displays median and IQR values, while Panel (**right**) shows individual data points.

No intergroup differences emerged for CD171+ vesicles, although NLGN3+ vesicle concentrations were significantly lower in the Alcohol group on day 1 (Figure 7). Moreover, the individual-value plots revealed a consistent trend toward lower concentrations in the Alcohol group throughout the entire observation period.

**Figure 8:**
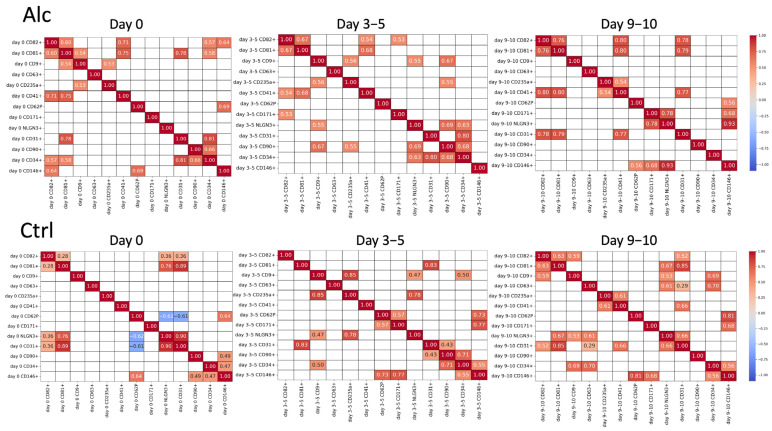
Correlation matrix of extracellular vesicle fractions. Alc—alcohol dependence group; Ctrl—healthy volunteers. Red denotes positive correlations, and blue denotes negative correlations. The figure shows the Spearman correlation results. Only significant (*p* > 0.05) correlations are displayed.

Overall, the concentrations of the distinct vesicle subsets were weakly intercorrelated in both the Alcohol group and the control group (Figure 8). However, the pattern of the most significant correlations differed markedly between the groups. Notably, throughout the entire observation period, the abundance of CD41+ vesicles was positively correlated with that of CD81+ and CD82+ vesicles in the Alcohol group. A strong positive correlation (ρ > 0.7) between CD31+ and CD81+ vesicles was observed throughout the period in both groups. Interestingly, CD62P+ vesicles consistently correlated with CD146+ vesicles over the entire observation period in the control group, whereas in the Alcohol group this association was weaker and less stable. Furthermore, CD34+ vesicle concentrations at the first and second time points consistently correlated with those of CD31+ and CD90+ vesicles. It is noteworthy that by the end of the observation period in the Alcohol group, NLGN3+ vesicles exhibited an extremely strong correlation with CD146+ vesicles (ρ = 0.93), which was not observed in the control group. Overall, the correlations in the control group appeared more random and did not persist over time, whereas the correlation pattern in the Alcohol group was more stable.

**Figure 9:**
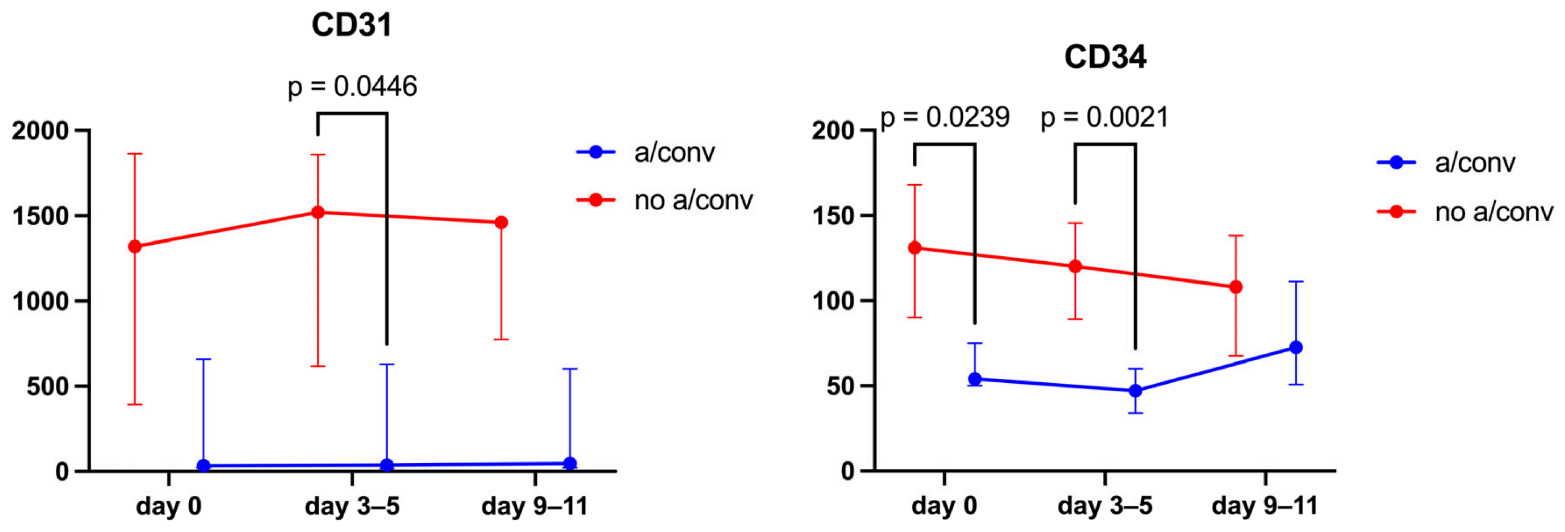
Concentrations of CD31+ and CD34+ vesicles in relation to anticonvulsant treatment. Blue points: median concentration in anticonvulsant-treated patients (*n* = 9); red points: median concentration in patients not taking anticonvulsants (*n* = 5). Vertical blue lines: IQR for anticonvulsant-treated patients; vertical red lines: IQR for patients not taking anticonvulsants.

We also conducted an additional post hoc exploratory analysis of the associations of concomitant medications and smoking status with vesicle concentrations. Smoking, antipsychotic use, and antidepressant use showed no significant differences in vesicle concentrations. Anticonvulsant use was associated with lower concentrations of CD31+ and CD34+ vesicles. After applying a mixed model and correcting for multiple comparisons, only the differences in CD34+ vesicles at the first time point and in both markers at the second time point remained significant (Figure 9). However, given the pronounced imbalance in subgroup sizes, these findings should be interpreted with caution.

**Figure A2:**
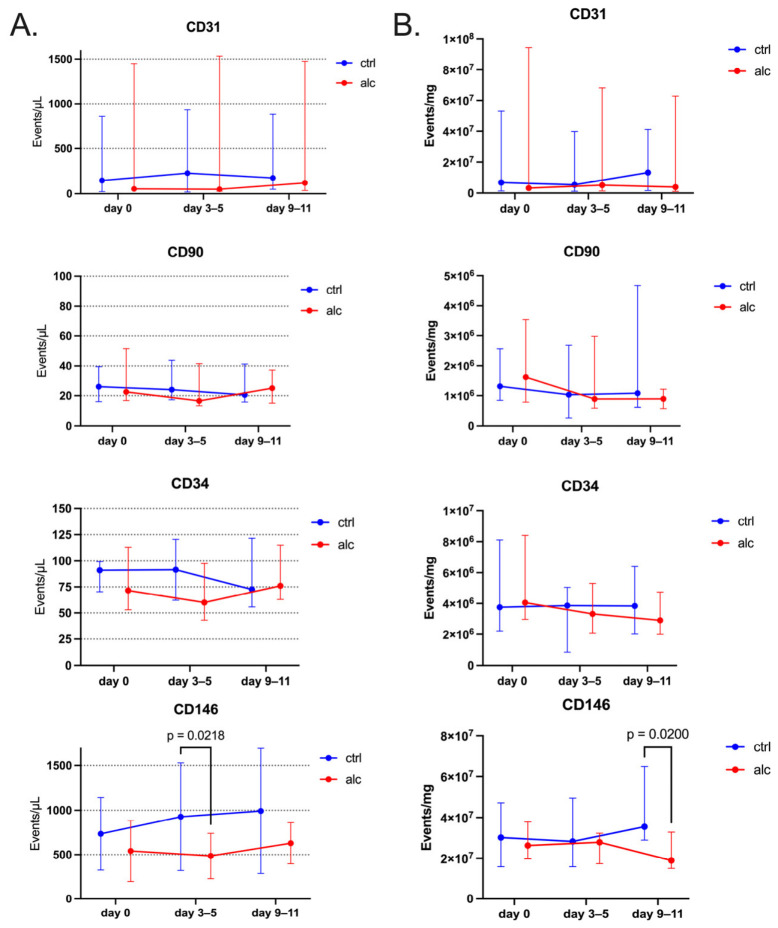
Concentrations of vesicles carrying platelet and erythrocytes markers. Blue points—median concentration in the control group. Red points—median concentration in the main group. Vertical blue lines—interquartile ranges in the control group. Vertical red lines—interquartile ranges in the Alcohol group. The upper part of the figure (**A**) corresponds to event counts expressed as events/μL, while the lower part (**B**) corresponds to counts normalized to total protein (events/mg).

**Figure A3:**
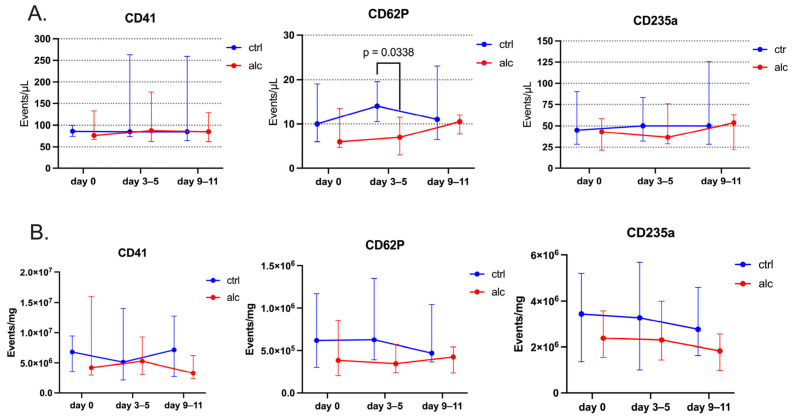
Concentrations of vesicles carrying endothelial markers. Blue points—median concentration in the control group. Red points—median concentration in the main group. Vertical blue lines—interquartile ranges in the control group. Vertical red lines—interquartile ranges in the Alcohol group. Panel (**A**) (**left**) shows event counts expressed as events/μL, while Panel (**B**) (**right**) shows counts normalized to total protein (events/mg).

**Figure A4:**
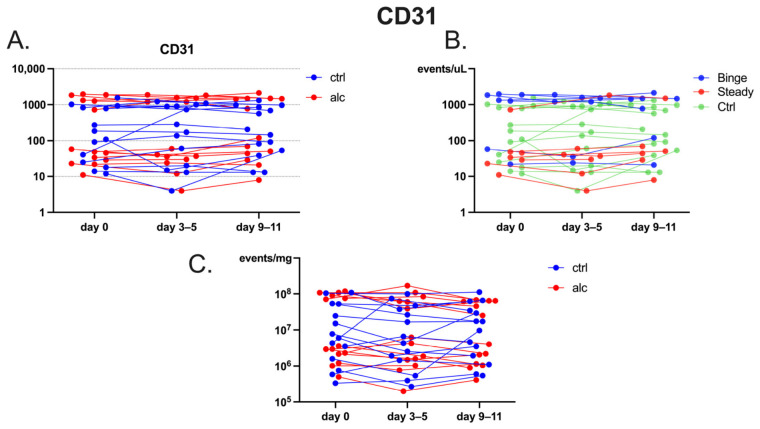
Concentrations of vesicles carrying CD31. (**Left**) graph: Blue points—individual values in the control group. Red points—individual values in the main group. (**Right**) graph: Blue points—individual values of patients with a recurrent pattern of use. Red points—individual values of patients with a constant pattern of use. Green points—individual values in the main group. Panels (**A**,**B**) show event counts expressed as events/μL, while Panel (**C**) shows counts normalized to total protein (events/mg).

**Figure A5:**
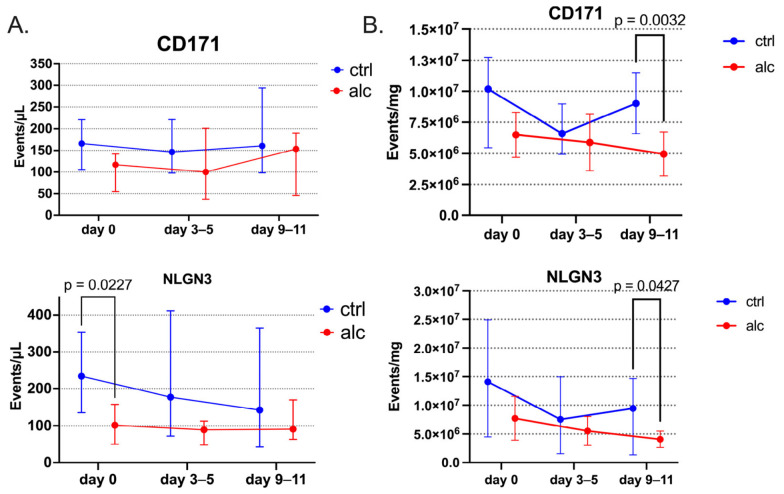
Concentrations of vesicles expressing neuronal markers. Blue points—median concentration in the control group. Red points—median concentration in the Alcohol group. Vertical blue lines—interquartile ranges in the control group. Vertical red lines—interquartile ranges in the main group. Panel (**A**) (**left**) shows event counts expressed as events/μL, while Panel (**B**) (**right**) shows counts normalized to total protein (events/mg).

**Figure A6:**
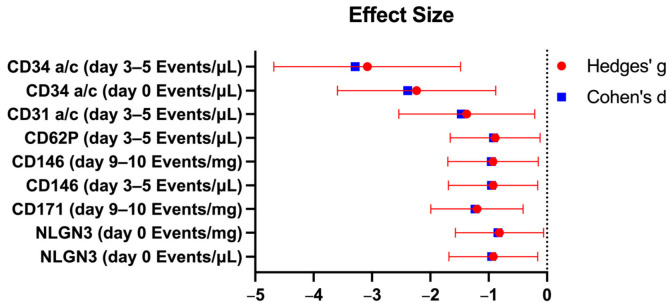
Observed effect sizes for all significant comparisons.

Finally, we performed a sensitivity analysis by adjusting for total protein (Appendix A Figure A2, Figure A3, Figure A4 and Figure A5). As a result, the set of markers exhibiting significant differences expanded to include CD171, while CD62P+ no longer showed significance; additionally, the time points at which differences were detected shifted. Effect sizes for all significant differences were large (Hedges’ g > 0.8) (Appendix A Figure A6). It should be noted, however, that total protein normalization is a suboptimal approach in this context, as protein content in plasma-derived SEC fractions may largely reflect co-isolated soluble proteins rather than true EV yield, potentially introducing ratio-related artifacts. Therefore, these protein-adjusted results should be interpreted with caution.

## 3. Discussion

In the present study, we isolated and characterized EVs from patients during acute alcohol withdrawal syndrome and from healthy volunteers over a 10-day period. Greater polydispersity and size heterogeneity of vesicles were detected in the Alcohol group by DLS and NTA. Furthermore, representative microscopy images revealed greater size diversity and the presence of vesicles with altered morphology. Using flow cytometry, we analyzed the concentrations of vesicle fractions expressing erythrocyte-, platelet-, endothelium-, or neuron-associated markers. EVs carrying NLGN3, a neuron-associated marker, were found at lower concentrations in patients at admission. Moreover, CD146+ and CD62P+ EVs exhibited lower concentrations at the 3–5 day time point in the Alcohol group compared with healthy volunteers. Furthermore, an exploratory post hoc analysis revealed that CD31+ EVs separated into two clusters: concentrations were higher in patients with a binge drinking pattern and markedly lower in those with a steady drinking pattern. In addition, the subgroup with lower CD31+ EV concentrations also exhibited reduced levels of other vesicles.

Many studies show that vesicle concentrations increase upon cell exposure to alcohol. It has been shown that in microglial cell cultures exposed to alcohol, the concentration of vesicles in the 100–200 nm range increases compared to controls [15]. Also, in a study on alcoholic hepatitis, vesicle concentration and size were greater in patients than in the control group [20]. Interestingly, using vesicles isolated from rodent stool, it was also shown that vesicles in the alcohol-treated group were morphologically different from controls and more abundant per field of view [21]. It is worth noting that several studies have demonstrated a link between both acute and chronic alcohol intoxication and sEV biogenesis as well as changes in lipid composition [15,16,17,22], which may lead to alterations in membrane characteristics such as flexibility, permeability, and density. Moreover, alcohol consumption may increase intestinal epithelial permeability and the entry of a greater number of bacterial vesicles into the circulating blood [23]. The morphological features observed in representative microscopy images warrant a more comprehensive analysis across multiple blinded fields; therefore, our findings should be regarded as exploratory. Data on the effects of pharmacological agents on EV morphology remain scarce. In the literature, we identified only isolated reports on the influence of anticancer drugs on vesicle morphology [24,25,26], and a single study that found no morphological differences following morphine administration [27]. This highlights a substantial gap in the understanding of how medications may alter EV characteristics, an issue that is particularly relevant in clinical cohorts where polypharmacy is common.

The discrepancy between the mean vesicle size determined by DLS (179.7 nm) and NTA (106.9 nm) is expected and stems from the fundamental differences in the measurement principles of the two techniques [2]. DLS measures the intensity-weighted hydrodynamic diameter, which is heavily biased toward larger particles because the scattering intensity scales with the sixth power of the particle diameter. In polydisperse samples, even a small number of larger vesicles or aggregates can substantially increase the reported mean size. NTA, in contrast, tracks individual particles and yields a number-weighted size distribution, which is less influenced by rare large events and more accurately reflects the predominant population of smaller vesicles.

When analyzing plasma extracellular vesicle fractions, we selected erythrocyte-associated (CD235a+) and platelet-associated (CD41+, CD62P+) EV markers. These subpopulations are not only the most abundant in plasma but, owing to their proinflammatory and procoagulant activities, may also contribute to the systemic responses triggered by alcohol consumption [28]. Chronic high-dose alcohol consumption possibly leads to endothelial dysfunction [29], accordingly, vesicles positive for endothelial cell-associated markers CD31, CD90, CD34, and CD146 were also included in the analysis. It has been shown that two months of alcohol consumption in mice reduces vascularization by inhibiting endothelial cell proliferation and inducing senescence [30] and reduces the number of vessels with high CD31 expression. It has also been shown that alcohol intoxication reduces the concentration of CD31+ vessels in the substantia nigra [31]. It has been shown that endothelial cells expressing CD31 in the alcohol-damaged region of the blood–brain barrier are associated with increased oligodendrogenesis and also with alcohol-seeking behavior [32]. Higher CD31 expression in the prefrontal cortex has also been shown to be associated with more severe behavioral symptoms of alcoholism in mice [33]. A recent study with multiplex analysis of vesicles in alcoholic liver disease showed that a higher level of CD31+ vesicles is associated with a better response to therapy and a more favorable prognosis [20]. These data are consistent with our results and may indirectly suggest that patients with reduced CD31+ EV concentrations consumed alcohol more heavily and in a steady pattern. It is also important to highlight the absence of significant abnormalities in routine blood tests, which might indicate a higher sensitivity of vesicular fractions to heavy alcohol use.

Given that addiction is primarily a psychiatric disorder, we also assessed the representation of neuron-associated markers. L1CAM is a transmembrane glycoprotein crucial for nervous system development, including neuronal migration, differentiation, and axon growth [34]. This protein is a conventional marker of neuronal EVs and is most commonly used for confirmation and affinity isolation of neuronal EVs [35]. NLGN3 is a postsynaptic cell adhesion molecule that acts as a synapse organizer, essential for excitatory and inhibitory synaptic transmission [36]. It is expressed in all cell types of the brain. Recently, it has also been demonstrated to be a suitable target for isolating neuronal EVs [37].

Classic [38,39,40] and some more recent studies [41] have shown that alcohol impairs the function of axonal growth cones via an L1CAM-dependent mechanism, and that an L1CAM-rich environment prevents this effect. Furthermore, L1CAM likely possesses binding sites to which alcohol directly binds [42]. It has also been shown that L1CAM protects cells from ethanol-induced death [43]. Ethanol has been demonstrated to reduce the enrichment of lipid rafts with L1CAM [44].

It has been shown that L1CAM+ EVs from depressed patients exert depressogenic effects in mice [45]. Concentrations of L1CAM+ EVs are reduced in primary progressive multiple sclerosis [46]. It has been shown that GFAP, but not L1CAM, is more strongly expressed in vesicles from individuals who drink [18]. No data exist regarding the interaction between NLGN3 and alcohol; however, evidence shows that NLGN3 protects neurons from ischemic injury [47]. NLGN3 mutations are associated with autism and cognitive impairment [48].

However, several limitations must be acknowledged. The primary limitation of our study is the inability to disentangle the effects of treatment, alcohol withdrawal, chronic intoxication, and unmeasured confounders on the parameters of interest. Nevertheless, we attempted to address this issue through a longitudinal design, given that at admission patients were treatment-naïve and in the earliest stages of alcohol withdrawal syndrome. The studied cohort was similar in size to those of previous studies on EVs in alcohol use disorders [9], but relatively small. Therefore, larger sample sizes are needed to confirm these findings. Furthermore, our study lacked functional and EV cargo assessments; consequently, insights into the role of these changes remain restricted. Another limitation is that, by using only a single SEC fraction, we may have missed some EV populations that elute in later fractions; however, this fraction was chosen to minimize protein contamination while retaining the highest vesicle yield. Additionally, it should be noted that the isolated vesicle samples were frozen and thawed prior to flow cytometry, which may have induced aggregate formation and skewed the absolute concentration values of vesicles. Nevertheless, all samples were processed and stored under identical conditions. Of note, we observed substantial interindividual and dynamic variability in the concentrations of distinct vesicle fractions among both healthy donors and patients. Using flow cytometry, a recent study of healthy donors exposed to psychological and physical stress demonstrated that within one hour following physical stress, the concentrations of CD9-, CD63-, and CD41-positive vesicles changed significantly, accompanied by considerable intragroup variability [49]. This suggests that difficult-to-control, unaccounted factors may have caused the redistribution of vesicle fractions.

In summary, our work demonstrates that EVs from patients with AWS exhibit distinct morphological features, size distribution, and concentration profiles, accompanied by high intragroup variability among vesicle subpopulations. Concentrations of EVs bearing NLGN3 and CD146+ EVs were lower in patients, whereas CD31+ EVs formed two distinct clusters, with higher levels in patients displaying a binge drinking pattern but markedly reduced levels in patients with a steady drinking pattern. In addition, the subgroup with lower CD31+ EV concentrations also exhibited reduced levels of other vesicles.

## 4. Materials and Methods

### 4.1. Participants

This study included 28 individuals: 14 patients with alcohol use disorder treated at the inpatient unit of the Moscow Research and Practical Center for Narcology, Moscow Department of Health, and 14 healthy volunteers.

Inclusion criteria for the Alcohol group were:

(1) Age 18 to 65 years.

(2) Diagnosis F10.3–Alcohol withdrawal state. The diagnosis was established based on ICD-10 criteria by a qualified psychiatrist upon the patient’s admission to the hospital.

When enrolling healthy volunteers, screening for risk of alcohol-related problems was performed using the RUS-AUDIT-S questioner, which has been validated for the Russian-speaking population [50]. The test contains three questions about the participant’s history of alcohol use. The total possible score ranges from 0 to 12.

Inclusion criteria for the control group were:

(1) Age 18 to 65 years.

Exclusion criteria for both groups were:

(1) Presence of any of the following disorders: ICD-10 F2x… (schizophrenia spectrum disorders); F3x… (mood disorders); history of ischemic stroke; chronic cardiovascular insufficiency; dementia of any origin; autoimmune diseases (including past history); oncological diseases (including past history); primary (non-alcohol-related) epilepsy; history of surgical interventions on the central nervous system; and chronic infectious disease.

(2) Pregnancy.

(3) An additional exclusion criterion for the control group was scoring in risk zones 2–4 (more than 1 point on the test for women and more than 3 points for men) according to the WHO-recommended interpretation of the RUS-AUDIT-S test.

Drinking pattern was classified as binge or steady in accordance with the criteria established by E. Epstein et al. [51].

### 4.2. Blood Collection

Blood samples were collected from patients three times: on day 1 of hospitalization, on days 3–5 of hospitalization, and on days 9–11. Blood samples from healthy volunteers were also collected three times at corresponding intervals: on the day of enrollment in the study, after 3–5 days, and after 9–11 days. Blood samples were collected after an overnight fast at approximately 10:00 a.m.

### 4.3. Sample Preparation

Six milliliters of venous blood was drawn from the cubital vein into vacuum tubes containing K3EDTA as an anticoagulant. The blood was centrifuged at 1500 *g* for 15 min. A 2.0 mL aliquot of the resulting plasma was transferred to a clean tube and centrifuged again at 1500 *g* for 15 min. Next, 1.5 mL of the supernatant was transferred to a new tube and centrifuged at 3000 *g* for 30 min, after which 1.4 mL of the obtained supernatant was centrifuged at 12,000 *g* for 30 min. After the final centrifugation, 1.0 mL of the supernatant was collected, frozen at −20 °C, and stored at the same temperature until further processing.

### 4.4. Isolation of Extracellular Vesicles from Plasma

A chromatography column was filled with 10 mL of Sepharose CL-2B resin (GE Healthcare, Chicago, IL, USA) and equilibrated with phosphate-buffered saline (PBS, tablets, Biolot, Saint Petersburg, Russia). Thawed plasma samples (0.5 mL) were loaded onto the column. After the sample had entered the column, 3.0 mL of PBS was immediately added, and the eluate was discarded. Next, another 1.0 mL of PBS was applied, and the eluate (1.0 mL) was collected for subsequent analysis. The isolated EV fraction was frozen at −20 °C and stored at the same temperature until needed. EVs were characterized according to the MISEV guidelines [2].

### 4.5. Transmission Electron Microscopy

To analyze isolated EV samples, a HRTEM Titan Themis Z (Thermo Fisher Scientific, Waltham, MA, USA) was used. A carbon 300 mesh Cu grid was treated by using Fischione model 1070 NanoClean system (E.A. Fischione Instruments, Inc., Export, PA, USA) to make the film hydrophilic. Then the grid was incubated with 6 μL of diluted 1:50 in DI water EV sample for 2 min. The excess of EVs sample was removed by blotting the grid perpendicular to the filter paper. The grid was then incubated in a drop of 1.5% uranyl acetate water solution for 45 s, followed by blotting the stain away using filter paper and letting the grid dry completely before imaging. The grid was fixed on the sample holder, and capsules were analyzed at magnification ranging from 10,000 to 100,000 times. CellProfiler™ Cell Image Analysis Software version 4.0 was used for processing HRTEM images of extracellular vesicles [52]. Representative microscopy images were acquired and utilized for analysis.

### 4.6. Total Protein Concentration Assesment

Protein concentration was assessed using the Bradford method. Samples were mixed with Coomassie G-250 dye in a 2:3 ratio in wells of a 384-well plate, incubated at room temperature for 15 min, and the absorbance at 595 nm was measured using a Hidex Sense plate spectrofluorometer (PerkinElmer, Turku, Finland). A calibration curve was constructed using bovine serum albumin over a concentration range of 0.001 mg/mL to 1 mg/mL.

### 4.7. Dynamic Light Scattering

Dynamic light scattering of EVs was measured using a Zetasizer Nano S instrument (Malvern Panalytical, Worcestershire, UK). A 100-μL aliquot of the sample was placed into a disposable polystyrene cuvette, and dynamic light scattering was measured in four replicates of 20 s each. The instrument allows calculation of nanoparticle size as well as determination of the scattered photon count, expressed in thousands of photons per second (kcps). The total number of scattered photons is used to calculate particle size but can also serve as an independent parameter characterizing the sample. All samples were analyzed.

### 4.8. Nanoparticle Tracking Analysis

Nanoparticle tracking analysis was performed using a NanoSight NS300 instrument (Malvern Panalytical, Worcestershire, UK). A laser with a wavelength of 488 nm was used as the light source. The sample was continuously delivered to the flow cell using a syringe pump, which allowed video recording of the movement of a large number of individual EVs (at least 2000 tracks per sample). Two videos of 30 s each were recorded. Subsequent computer processing of the recorded videos enables tracking of the movement trajectory of each individual particle and calculation of particle size. During the experiment, the sample temperature was maintained at 25 °C. A total of nine samples, obtained from three participants per group, were analyzed after a 1:50 dilution.

### 4.9. Immunoblotting

All samples (1 mL each) were precipitated with TCA (trichloroacetic acid). The pellets were boiled in 1× loading buffer, loaded onto 10% polyacrylamide gels, and electrophoresis was performed according to Laemmli. Proteins were transferred onto nitrocellulose membranes by wet transfer. Non-specific binding sites on the membrane were blocked in 5% non-fat dry milk solution for 60 min at room temperature with agitation. Primary antibodies (diluted 1:1000 in 5% milk) were incubated overnight at 4 °C with agitation. Membranes were washed to remove excess primary antibodies and then incubated with the corresponding horseradish peroxidase-conjugated secondary antibody solution for 60 min at room temperature with agitation. Excess secondary antibodies were washed off, and membranes were developed using SuperSignal™ West Femto Maximum Sensitivity Substrate (#34095, Thermo Scientific, Waltham, MA, USA) with a MicroChemi 4.2 instrument (DNR, Jerusalem, Israel). The resulting images were analyzed using GelQuant Express Analysis version 3.1 software. Antibodies used were against Alix (Alix (E4T7U) Rabbit Monoclonal Antibody #18269) and against Flotillin-1 (Flotillin-1 (D2V7J) Rabbit Monoclonal Antibody #18634) from Cell Signaling Technology, Danvers, MA, USA. A goat anti-rabbit IgG (H+L)–HRP conjugate (Bio-Rad, #1706515, Hercules, CA, USA) secondary antibody was used at a 1:5000 dilution. Representative immunoblot images were acquired.

### 4.10. Immunophenotyping of Extracellular Vesicles

**Figure A7:**
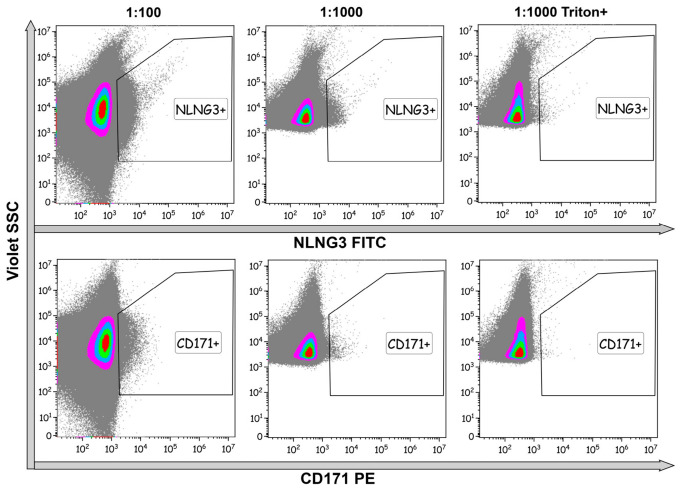
Representative dot plots of the results of EVs samples immunostaining with anti-NLNGN3 and anti-CD171 monoclonal fluorescent antibodies. Results presented in 1:100 and 1:1000 dilutions. The rightmost column demonstrates the results of detergent plus control. Pseudo-color dot plots reflect relative vesicles expression density.

The phenotyping of circulating EVs followed the official guidelines of the International Society for Extracellular Vesicles [53,54] and was carried out as previously described by our group [55,56,57,58]. Immunofluorescence staining was performed using the antibody panels listed in Appendix A Table A1. Representative dot plots of results of EVs staining with NLNG3 and CD171 in different dilutions and after detergent treatment are presented in the Appendix A Figure A7.

All five panels were prepared and tested separately in dedicated tubes. For each sample, 100 µL of the specimen was incubated with 1 µL of each monoclonal antibody for 25 min at 20 °C in the dark. Single-stained controls were used to assess potential spectral overlap between fluorochromes and to validate compensation settings. Isotype controls included: Alexa Fluor 488 Mouse IgG1, κ Isotype Ctrl (BioLegend Inc., San Diego, CA, USA, clone: MOPC-21, cat. 400129, 200 µg/mL); PE Mouse IgG1, κ Isotype Ctrl (BioLegend Inc., clone: MOPC-21, cat. 400114, 200 µg/mL); and APC Mouse IgG1, κ Isotype Ctrl (BioLegend Inc., clone: MOPC-21, cat. 400122, 200 µg/mL). All isotype and single-stained controls were processed and measured under the same conditions and at the same concentrations as the corresponding fluorochrome-conjugated antibodies.

For each stained panel, a serial dilution procedure was performed [57,59]. Briefly, 20 µL of the stained sample was mixed with 180 µL of PBS (Biolot, St. Petersburg, Russia) to obtain a 10-fold dilution. This dilution was further serially diluted by adding 100 µL of sample to 900 µL of PBS. For each panel, at least three dilutions (ranging from 10-fold to 1000-fold) were analyzed, and all dilutions were used to calculate the initial EV concentration.

A buffer-only control (PBS) and a reagent control (0.5 µL of each monoclonal antibody in 50 µL PBS) were acquired using the same flow cytometer settings (triggering threshold, voltages, flow rate) as the samples. To enable comparisons with serially diluted stained samples, both controls were diluted five-fold with PBS in the same manner as the samples. The event rate of the reagent control was comparable to that of the buffer-only control. Unstained controls were also measured under identical conditions and dilutions as the stained samples. Flow cytometer acquisition settings (including threshold, voltages, and flow rate) remained unchanged for all controls and stained samples.

To avoid coincidence events, a working dilution was established for each staining panel. The optimal dilution was defined by the following criteria: a linear relationship between the dilution factor and the event count, and no differences in fluorescence or scatter intensity among events. The optimal dilution for each panel was subsequently used to calculate EV concentrations, reported as events per µL.

EV phenotyping was performed on a Cytoflex S high-sensitivity flow cytometer (Beckman Coulter, Brea, CA, USA) as previously described [57,59]. The trigger was set on the 405 nm laser with a threshold of 2000 arbitrary units. Instrument calibration was carried out using the Cytometry Sub-Micron Particle Size Reference Kit (Molecular Probes, Life Technologies, Eugene, OR, USA) and Megamix-Plus FSC and Megamix-Plus SSC beads (Biocytex, Marseille, France), which include FITC-labeled reference beads ranging from 100 to 1000 nm. The flow rate was set to 120 µL/min. Stained particles were detected using side scatter from the violet 405 nm laser and appropriate fluorescence channels.

To confirm the membrane nature of the detected objects, additional detergent controls were employed. Samples were first stained with monoclonal antibodies and phenotyped as described above. Then, equal volumes of either PBS or 2% Triton X-100 in PBS were added to the stained samples and incubated for 20 min at room temperature in the dark, followed by flow cytometry analysis. A reduction of at least 90% in event count after detergent treatment indicated membrane lysis, confirming that the detected events were indeed EVs. 

Data analysis was performed using CytExpert v.2.4 (Beckman Coulter, Brea, CA, USA) and Kaluza 2.1 (Beckman Coulter, Brea, CA, USA) software. EV concentrations are presented as the number of events positive for the respective markers per microliter.

### 4.11. Statistical Analysis

Quantitative variables were assessed for normality of distribution using the Shapiro–Wilk test. Quantitative data were described using the mean (M) and standard deviation (SD) for normally distributed data, or the median (Me) and interquartile range (Q1–Q3) for non-normally distributed data. For comparing two groups on a quantitative variable that followed a normal distribution in each group, with equal variances assumed, Student’s *t*-test was used. For comparing two groups on a quantitative variable that deviated from a normal distribution, with equal variances assumed, the Mann–Whitney U test was applied. Comparisons of interval values were performed using two-way analysis of variance (two-way ANOVA) or mixed-effects analysis. Multiple comparisons were corrected for using Tukey’s honestly significant difference test. Differences were considered statistically significant at *p* < 0.05. Statistical analyses were carried out using StatTech, version 4.8.5 (StatTech, Kazan, Russia).

## Figures and Tables

**Table 1:** Characteristics of the sample cohort.

Characteristics	Alcohol	Healthy Volunteers
Sex (M/F)	5/9	5/9
Age, M ± SD	44.6 ± 8.3	44.1 ± 6.6
BMI, M ± SD	25.8 ± 2.4	23.4 ± 3.5
Smoking status (yes), *n* (%)	12 (85.7)	3 (21.4)
CIWA-Ar, M ± SD	13.4 ± 3.1	
Age of the onset of alcohol withdrawal syndrome (AWS), years Me [IQR]	34.00 [30.00; 35.75]	
Years since onset of AWS, Me [IQR]	12.50 [8.25; 15.00]	
Age at onset of regular alcohol use, years Me [IQR]	26 [20; 31]	
Years from onset of regular use to AWS onset, years Me [IQR]	6.00 [4.25; 10.00]	
Daily tolerance to alcohol, liters Me [IQR]	0.50 [0.50; 1.00]	
Duration of alcohol use prior to admission, days Me [IQR]	14.00 [5.50; 26.50]	
Amount of last alcohol consumption prior to admission, liters Me [IQR]	0.30 [0.25; 0.50]	
Alcoholic hepatitis, *n* (%)	8 (57.1)	
Alcoholic fatty liver disease (AFLD), *n* (%)	3 (21.4)	

BMI—Body mass index; CIWA-Ar—Clinical Institute Withdrawal Assessment for Alcohol, Revised. Main quantitative characteristics of alcohol dependence and comparability with the control group in terms of age, sex, and BMI are shown in the table.

**Table 2:** Admission blood test results of the Alcohol group.

Parameters	Mean ± SD/Median	95% CI/ Q_1_–Q_3_	Min	Max	Reference Intervals
Red blood cells (RBC), 10^12^/L M ± SD	4.34 ± 0.46	4.08–4.61	3.80	5.48	4.0–6.0
Platelets (PLT), 10^9^/L M ± SD	201.14 ± 93.25	147.30–254.99	32.00	340.00	130–400
White blood cells (WBC), 10^9^/L M ± SD	7.15 ± 2.04	5.98–8.33	3.86	11.40	4.0–9.0
Monocytes (MON), 10^9^/L M ± SD	0.50 ± 0.23	0.37–0.63	0.21	0.97	0.2–1.0
Lymphocytes (LYM), 10^9^/L Me	2.08	1.80–2.70	0.50	37.80	0.8–4.8
Neutrophils (NEU), 10^9^/L M ± SD	4.46 ± 1.76	3.44–5.48	1.65	7.70	1.5–6.9
erythrocyte sedimentation rate (ESR), mm/h M ± SD	19.07 ± 10.49	13.01–25.13	5.00	41.00	1.0–10.0
ALT (alanine aminotransferase), U/L Me	30.35	27.47–55.92	17.10	256.00	5.0–45.0
AST (aspartate aminotransferase), U/L Me	38.98	37.05–55.90	17.00	242.00	5.0–40.0
GGT (gamma-glutamyl transferase), U/L Me	56.05	38.23–133.95	22.00	476.00	5.0–50.0
Total protein, g/L M ± SD	71.03 ± 6.91	67.04–75.02	56.25	81.20	64.0–83.0

The table presents the results of blood analysis of patients at admission. Reference intervals are indicated according to the manufacturer’s recommendations.

**Table 3:** Comparative analysis of groups with “low” and “high” concentrations of CD31+ vesicles.

Parameter	CD31+ <700 (*n* = 8)	CD31+ >700 (*n* = 6)	*p*-Value
Age, M (SD)	45.65 (7.26)	42.45 (7.53)	0.273
BMI, M (SD)	25.50 (3.20)	23.32 (3.00)	0.083
d0 Vesicular Protein, Me [IQR]	0.02 [0.01; 0.03]	0.01 [0.01; 0.02]	0.466
d3-5 Vesicular Protein, Me [IQR]	0.02 [0.01; 0.03]	0.02 [0.02; 0.03]	0.410
d9-11 Vesicular Protein, Me [IQR]	0.02 [0.02; 0.03]	0.02 [0.02; 0.03]	0.760
d0 CD82+, Me [IQR]	22.00 [18.00; 42.00]	54.00 [37.50; 77.00]	0.029 *
d0 CD81+, Me [IQR]	97.00 [29.00; 143.00]	868.00 [761.50; 980.50]	<0.001 *
d0 CD9+, M (SD)	417.71 (261.22)	1194.73 (1131.98)	0.047 *
d3-5 CD82+, M (SD)	25.00 (18.65)	50.82 (31.43)	0.011 *
d3-5 CD81+, Me [IQR]	66.00 [34.00; 224.00]	741.00 [577.00; 873.50]	<0.001 *
d9-11 CD82+, Me [IQR]	23.00 [10.00; 36.00]	46.00 [34.50; 80.00]	0.002 *
d9-11 CD81+, Me [IQR]	62.00 [41.00; 164.00]	815.00 [662.00; 973.00]	<0.001 *
d0 CD41+, Me [IQR]	74.50 [65.25; 81.50]	99.00 [80.00; 520.00]	0.003 *
d3-5 CD41+, Me [IQR]	78.50 [60.75; 87.75]	171.00 [89.50; 330.00]	0.004 *
d9-11 CD41+, Me [IQR]	66.00 [61.00; 80.75]	127.00 [97.00; 393.00]	<0.001 *
d9-11 NLGN3+, Me [IQR]	74.00 [33.00; 157.00]	210.00 [101.00; 320.50]	0.029 *
d0 CD31+, Me [IQR]	34.00 [22.00; 58.00]	1265.00 [881.50; 1699.00]	<0.001 *
d0 CD34, M (SD)	68.29 (27.12)	110.64 (37.59)	0.002 *
d3-5 CD31+, Me [IQR]	36.00 [15.00; 61.00]	1198.00 [999.50; 1547.00]	<0.001 *
d3-5 CD34, M (SD)	61.65 (41.15)	105.91 (31.15)	0.005 *
d9-11 CD31+, Me [IQR]	52.50 [27.00; 99.00]	1310.00 [913.00; 1475.00]	<0.001 *
Age at onset of AWS, M (SD)	36.38 (6.30)	30.33 (5.13)	0.080
Years since onset of AWS, M (SD)	11.12 (4.70)	10.50 (6.95)	0.844
Age at regular use, M (SD)	27.75 (7.94)	23.50 (7.06)	0.320
Years from regular use to AWS, M (SD)	8.62 (4.93)	6.83 (3.87)	0.477
Tolerance (L), Me [IQR]	0.50 [0.50; 1.00]	0.50 [0.50; 0.88]	0.772
Duration of use before hospitalization (days), Me [IQR]	14.00 [8.25; 37.75]	12.00 [5.50; 15.50]	0.603
Amount of last use (L), M (SD)	0.33 (0.15)	0.36 (0.17)	0.757
RBC, 10^12^/L M (SD)	4.45 (0.53)	4.20 (0.35)	0.332
PLT, 10^9^/L M (SD)	214.75 (97.83)	183.00 (92.31)	0.550
WBC, 10^9^/L M (SD)	7.27 (2.18)	6.99 (2.02)	0.810
Total protein, M (SD)	70.90 (5.00)	71.21 (9.44)	0.938
ALT, U/L Me [IQR]	33.80 [27.85; 58.90]	28.05 [23.03; 49.55]	0.519
AST, U/L Me [IQR]	50.75 [35.00; 58.08]	37.80 [37.05; 38.82]	0.366
GGT, U/L Me [IQR]	53.15 [43.67; 60.38]	146.85 [33.33; 299.52]	0.459
Sex (f), *n* (%)	5 (62.5%)	4 (66.7%)	1.0
Binge drinking pattern, *n* (%)	2 (25.0%)	5 (83.3%)	0.103
Steady drinking pattern, *n* (%)	6 (75.0%)	1 (16.7%)	
Hepatic steatosis (yes), *n* (%)	6 (75.0%)	5 (83.3%)	1.0
Toxic hepatitis (yes), *n* (%)	3 (37.5%)	3 (50.0%)	1.0

Table presents the results of an exploratory analysis of clinical characteristics and vesicle concentrations according to CD31+ vesicle concentration subgroups. Comparisons were performed using the *t*-test or the Mann–Whitney U test for continuous variables, and Fisher’s exact test for categorical variables, without correction for multiple comparisons. * *p* < 0.05.

## Data Availability

The raw data supporting the conclusions of this article are available from the corresponding author upon reasonable request.

## Abbreviations

The following abbreviations are used in this manuscript:

ALT	alanine aminotransferase
AST	aspartate aminotransferase
AWS	Alcohol withdrawal syndrome
BMI	Body Mass Index
CD	cluster of differentiation
CNS	central nervous system
DLS	Dynamic light scattering
ESR	erythrocyte sedimentation rate
EV	extracellular vesicles
GGT	gamma glutamyl transferase
ICD	International Classification of Diseases
L1CAM	L1 cell adhesion molecule
LYM	lymphocytes
MON	monocytes
NEU	neutrophils
NLGN 3	neuroligin 3
NTA	Nanoparticle Tracking Analysis
PBS	phosphate-buffered saline
PLT	platelets
RBC	red blood cells
TCA	trichloroacetic acid
HRTEM	Transmission electron microscopy
WBC	white blood cells
WHO	World Health Organization

## Appendix A

**Table A1:** Panels of monoclonal antibodies for immunophenotyping of EVs.

Antibody	Manufacture	Receptor Specification	Cell Origin
Panel 1			
CD235a PE	BioLegend, San Diego, CA, USA	glycophorin A	erythroid precursors and erythrocytes
CD41 AF488	BioLegend, San Diego, CA, USA	α subunit of the gpIIb/IIIa (CD41/CD61) complex	platelets and megakaryocytes
Panel 2			
CD41 AF488	BioLegend, San Diego, CA, USA	α subunit of the gpIIb/IIIa (CD41/CD61) complex	platelets and megakaryocytes
CD62P PE	BD Pharmingen, Franklin Lakes, NJ, USA	type I transmembrane glycoprotein; P-selectin; platelet activation-dependent granule membrane protein (PADGEM); GMP-140	activated platelets, megakaryocytes, and endothelial cells
Panel 3			
CD31 FITC	BioLegend, San Diego, CA, USA	platelet endothelial cell adhesion molecule-1 (PECAM-1)	monocytes, platelets, granulocytes, endothelial cells and lymphocyte subsets
CD34 PE/Dazzle	BioLegend, San Diego, CA, USA	type I monomeric sialomucin-like glycophosphoprotein	hematopoietic stem/progenitor cells, bone marrow stromal cells, capillary endothelial cells, embryonic fibroblasts, some nervous tissue
CD90 PE/Cy7	BioLegend, San Diego, CA, USA	GPI-anchored protein, Thy-1	neuronal cells, a subset of CD34+ cells, fibroblasts, activated endothelial cells
CD146 PE	BioLegend, San Diego, CA, USA	integral transmembrane glycoprotein that belongs to the immunoglobulin superfamily	epithelial cells, endothelial cells, fibroblasts, activated T cells, multipotent mesenchymal stromal cells, and activated keratinocytes
Panel 4			
CD82 PE	BioLegend, San Diego, CA, USA		lymphoid tissues, glandular epithelia and islets of Langerhans; vesicles
CD81 FITC	BioLegend, San Diego, CA, USA		Microglia, astrocytes, tissue stroma with additional high levels in seminiferous ducts and lymphoid germinal center cells
CD9 PE/Cy7	BioLegend, San Diego, CA, USA		Vesicles, plasma membrane various cells
CD63 APC	BioLegend, San Diego, CA, USA		hematopoietic cells, endosomes, multivesicular bodies, vesicles
Panel 5			
CD171 PE	BioLegend, San Diego, CA, USA	L1 cell adhesion molecule	Cytoplasmic expression mainly in central nervouse system CNS, peripheral nervous system PNS and distal renal tubules.
NLGN3 FITC	Novus Biologicals, Centennial, CO, USA	Neuroligin 3	neuronal cells

The adjustment for protein yield based on the measured total vesicular protein concentrations in the samples was calculated using the formula:

(events/μL × 1000)/(mg/mL) = events/mg. (A1)
